# Intensive Care Unit-Acquired Bacteremia in Mechanically Ventilated Patients: Clinical Features and Outcomes

**DOI:** 10.1371/journal.pone.0083298

**Published:** 2013-12-23

**Authors:** Hsin-Kuo Ko, Wen-Kuang Yu, Te-Cheng Lien, Jia-Horng Wang, Arthur S. Slutsky, Haibo Zhang, Yu Ru Kou

**Affiliations:** 1 Institute of Physiology, School of Medicine, National Yang-Ming University, Taipei, Taiwan, ROC; 2 Department of Respiratory Therapy, Taipei Veterans General Hospital, Taipei, Taiwan, ROC; 3 The Keenan Research Centre, Li Ka Shing Knowledge Institute of St. Michael's Hospital, Toronto, Ontario, Canada; 4 Interdepartmental Division of Critical Care Medicine, University of Toronto, Toronto, Ontario, Canada; 5 Department of Anesthesia, Department of Physiology, Interdepartmental Division of Critical Care Medicine, University of Toronto, Toronto, Ontario, Canada; 6 Institute of Emergency and Critical Care Medicine, School of Medicine, National Yang-Ming University, Taipei, Taiwan, ROC; Kaohsiung Chang Gung Memorial Hospital, Taiwan

## Abstract

Intensive care unit (ICU)-acquired bacteremia (IAB) is associated with high medical expenditure and mortality. Mechanically ventilated patients represent one third of all patients admitted to ICU, but the clinical features and outcomes in mechanically ventilated patients who develop IAB remain unknown. We conducted a 3-year retrospective observational cohort study, and 1,453 patients who received mechanical ventilation on ICU admission were enrolled. Among patients enrolled, 126 patients who had developed IAB ≧48 hours after ICU admission were identified. The study patients were divided into IAB and no IAB groups, and clinical characteristics of IAB based on specific bacterial species were further analyzed. The multivariate Cox regression analysis showed that ventilator support for chronic obstructive pulmonary disease and congestive heart failure, and patients admitted from nursing home were the independent risk factors for developing IAB. Patients with IAB were significantly associated with longer length of ICU stay, prolonged ventilator use, lower rate of successful weaning, and higher rate of ventilator dependence and ICU mortality as compared to those without IAB. IAB was the independent risk factor for ICU mortality (HR, 1.510, 95% CI 1.054–1.123; p = 0.010). The clinical characteristics of IAB related to specific bacterial species included IAB due to *Pseudomonas aeruginosa* being likely polymicrobial, lung source and prior antibiotic use; *Escherichia coli* developing earlier and from urinary tract source; methicillin-resistant *Staphylococcus aureus* related to central venous catheter and multiple sets of positive hemoculture; and *Elizabethkingia meningoseptica* significantly associated with delayed/inappropriate antibiotic treatment. In summary, IAB was significantly associated with poor patient outcomes in mechanically ventilated ICU patients. The clinical features related to IAB and clinical characteristics of IAB based on specific bacterial species identified in our study may be utilized to refine the management of IAB.

## Introduction

Nosocomial infection is one of the leading causes of death and is associated with high medical expenditure [1]–[5]. Among nosocomial infections in the ICU, it has been reported that ICU-acquired bacteremia (IAB) contributes to an approximately 35% mortality rate [5]–[8]. Notably, critically ill patients undergoing mechanical ventilation (MV) represent one third of all patients admitted to ICU [9], but the clinical features and outcomes in mechanically ventilated patients who develop IAB have not yet been investigated. Furthermore, the influence of IAB on ventilator outcomes including successful weaning of ventilator, ventilator days and ventilator dependence remains unknown.

A major challenge at bedside is that intensivists may not have sufficient information to identify ICU patients at the risk of developing IAB and to distinguish the definite bacterial species prior to prescription of appropriate antibiotics. Although some studies report the predisposing factors for developing IAB [6], [10], it is uncertain whether the information originated from critically ill patients with and without MV can be applicable to mechanically ventilated patients and to refine the management of IAB. A number of studies also investigate nosocomial bacteremia in critically ill patients and focus on specific bacterial species [11]–[15]. These studies report clinical features related to one of several specific bacterial species and offer the information for intensivists to refine the management of IAB. However, the lack of comparative analysis among several bacterial species still leads to a difficulty for intensivists in differentiating the types of pathogens prior to prescription of appropriate antibiotics. The clinical characteristics of IAB related to specific bacterial species is critical for the management of IAB and may help improve the quality of care for ICU patients.

The aims of the study were two-fold: 1) To describe the clinical features of IAB in mechanically ventilated ICU patients, and to define the influence of IAB on patient outcomes; and 2) To identify the risk factors for developing IAB and to describe the clinical characteristics of IAB based on bacterial species. The results of our study might be utilized by intensivists to identify mechanically ventilated ICU patients who are at the risk of IAB and to refine the management of IAB based on clinical characteristics of specific bacterial species.

## Materials and Methods

### Ethics statement

We consulted with the institutional review board (IRB) of Taipei Veterans General Hospital, and informed consent was waived under the approval of our IRB according to the institutional guideline for a retrospective observational study. The study was registered at the IRB of Taipei Veterans General Hospital (IRB 97-08-24A), and the date of approval was September 4, 2008.

### Design, setting and patients

The retrospective observational cohort study was designed in January 2008 and was conducted in a 35-bed adult respiratory ICU in Taipei Veterans General Hospital, a tertiary teaching hospital in northern Taiwan. Patients were admitted from home, nursing home, local hospital, ordinary ward and other ICU for intensive critical care and/or the discontinuation of mechanical ventilator. All records of consecutive patients admitted to the ICU between July 1, 2006 and June 30, 2009 were retrospectively reviewed. Patients who received mechanical ventilation on ICU admission were enrolled and patients were excluded from the study if they 1) received only noninvasive positive pressure ventilation or oxygen therapy on ICU admission, 2) received invasive mechanical ventilation for less than 72 h after ICU admission, 3) bacteremia had developed within 48 h of ICU admission, or if 4) fungus infection was identified in blood culture.

### Measurements

The patient database collection included age, gender, comorbidities, APACHE II score [16], reasons for mechanical ventilation, sources of patients, ICU days, ventilator days, ICU mortality, and ICU discharge deposition. Blood culture and culture from a suspected source (lung, urine, intraabdominal site, soft tissue, catheter or other) were routinely carried out if a patient's temperature was >38.0°C or when infection was suspected on clinical grounds. Following a standard sterile procedure, two sets of blood culture were obtained from peripheral sites at a 30-min interval. A third blood sample for culture was collected if a central venous catheter (CVC) was in place. Blood samples were processed, and the identifications of microorganism were done by the BacT/Alert System (bioMérieux, Marcy-l'Etoile, France). Susceptibility testing was done according to the national standards regarding selection of antibiotics for antibiogram. If multiple episodes of bacteremia were observed during ICU stay, only the first episode was reviewed and recorded.

### Definitions

The definition of bacteremia episode is referred to as a positive isolation of a pathogenic bacterium from blood culture. Primary bacteremia was defined as a microorganism positive in blood culture with unknown infection source. Secondary bacteremia was defined as the same microorganism discovered in blood culture and in the identified source. IAB was defined as a bacteremia developing at 48 hours or later after ICU admission. The onset of IAB episode was defined as the time when a positive culture sample was collected. To identify skin pathogens such as coagulase-negative staphylococci, at least two sets of isolation were required to confirm the development of IAB that displayed the necessity for antibiotic treatment [10]. Polymicrobial bacteremia was defined as multiple pathogens being identified from a single set or different sets of blood culture within 48 hours [17]. Time delay for appropriate antibiotic was defined as the time gap between the onset of IAB and the administration of appropriate antibiotics. Prior antibiotic use was defined as any antibiotic prescribed within 14 days prior to confirmation of IAB. Catheter-related IAB was defined as the same microorganism discovered in the tip of the central venous or arterial catheter with at least 10^3^ colony-forming units and in blood culture. Antibiotic therapy was defined as appropriateness if antibiotic was active in vitro against the causative microorganisms based on the result of antimicrobial susceptibility test and when the route of administration and dosage conformed to current medical standards. An inappropriate antibiotic therapy was termed otherwise.

Sepsis was defined as the presence of infection together with systemic manifestations of infection, including temperature ≧ 38°C or ≤36°C, heart rate ≧90 beats/min, respiratory rate ≧ 20 breaths/min, white blood cells ≧ 12000/mm^3^ or ≤4000/mm^3^ or >10% premature bands [18]. The presence of shock was defined as a systolic blood pressure of less than 90 mmHg despite adequate fluid resuscitation and vasopressor use for more than 48 hours. Sepsis-induced organ dysfunction was defined as the following: 1) pulmonary dysfunction, PaO_2_/FiO_2_<300; 2) renal dysfunction, an increase serum creatinine level by 0.5 mg/dL from baseline or an absolute creatinine level of 2.0 mg/dL; 3) hepatic dysfunction, total bilirubin level >4 mg/dl; 4) coagulopathy, the presence of disseminated intravascular coagulation, prothrombin time (PT)>1.5 INR or activated partial thromboplastin time >60 seconds; 5) hematological dysfunction, a platelet count <100,000/mm^3^; and 6) neurological dysfunction, altered conscious level with a decreased Glasgow Coma scale [18]. Successful weaning was defined as ventilator-free time for more than 72 hours after the discontinuation of mechanical ventilation.

### Statistical analysis

SPSS software (version 15.0; SPSS Inc., Chicago, IL) was used for all analyses. Continuous variables were compared using the Student *t-*test for normally distributed variables and the Mann–Whitney U test and the Kruskal-Wallis test for non-normally distributed variables. Pearson's chi-square or Fisher's exact test was performed to compare categorical variables. A multivariate Cox regression model performed by the method of forward stepwise technique was applied using the variables associated with IAB and ICU mortality as a univariate analysis. All continuous data were presented as means ± standard deviations or medians with interquartile ranges. A p value <0.05 was considered statistically significant, and all p values were two-sided.

## Results

### Baseline characteristics of the study patients

**Figure 1:**
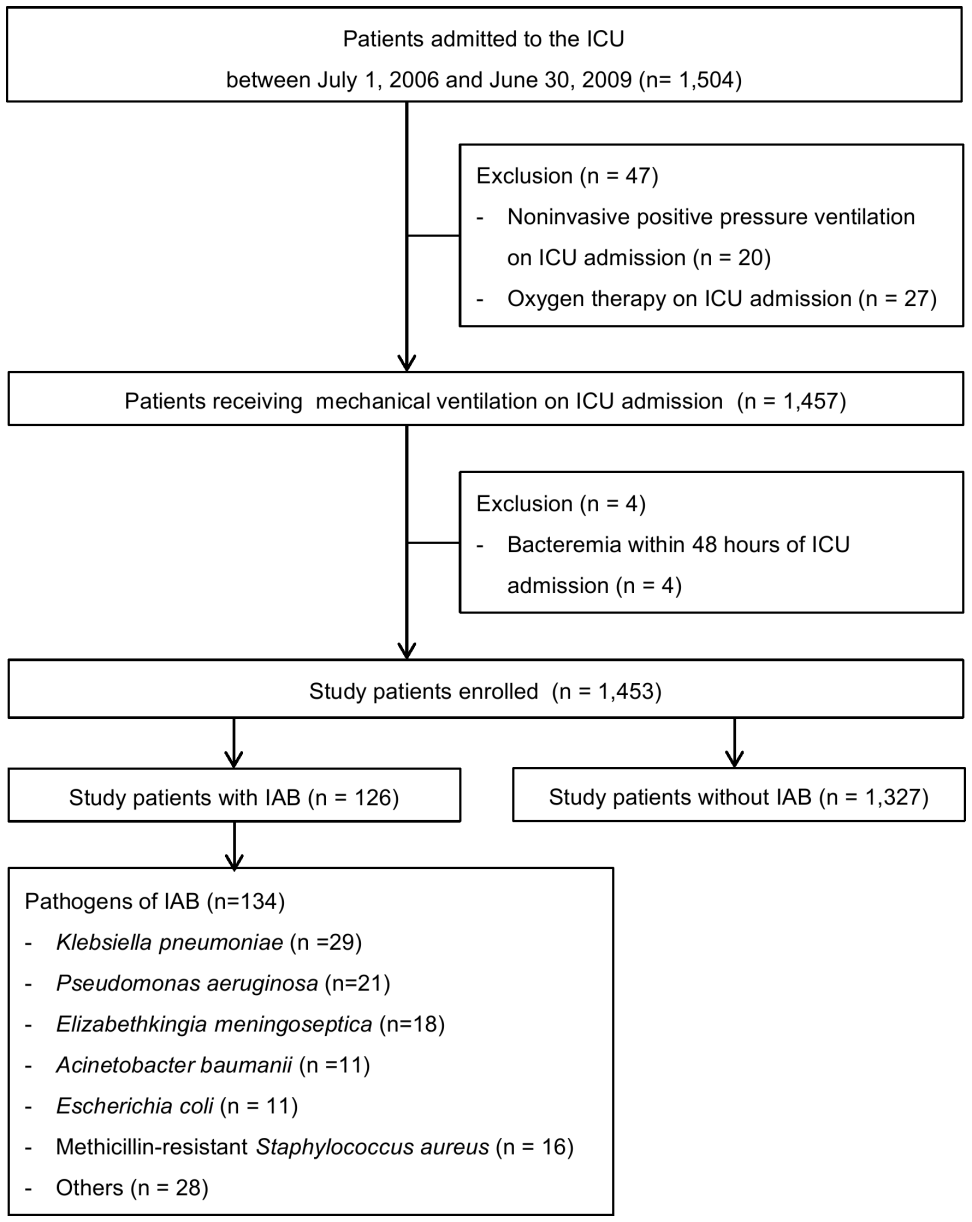
Flowchart of the study protocol. IAB, intensive care unit-acquired bacteremia.

**Figure 2:**
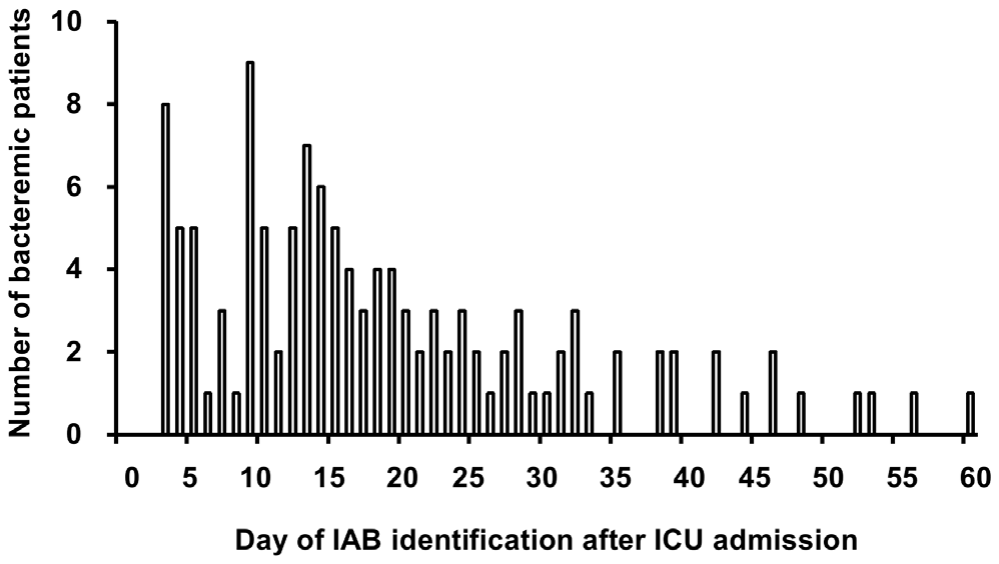
The mode of IAB development based on the numbers of IAB patients and the day of IAB identification after ICU admission.

A total of 1,504 patients were admitted to the ICU during the study period. Forty-seven patients were excluded who received noninvasive positive pressure ventilation (n = 20), and who only received oxygen therapy on ICU admission (n = 27). A total of 1,457 patients who received MV on ICU admission were reviewed. Among these patients, 4 patients who developed bacteremia within 48 hours of ICU admission were excluded. Thus 1,453 patients receiving MV on ICU admission were enrolled in the study (**Figure 1**). The median age and mean APACHE II score on ICU admission of study patients were 80 (76–85) years and 18.8 (±5.4), respectively. Overall ICU mortality was 15.8% (230/1453). Among the study patients, 126 (8.7%, 126/1453) bacteremic patients developed IAB after a median of 16 days (10–28) after ICU admission (**Figure 2**) and a median of 27 days (13–41) after initiating MV. A total of 134 pathogens associated with the first episode of IAB were depicted in **Table 1**, which included gram-negative bacteria recovered in 111 episodes (82.8%, 111/134) and gram-positive bacteria recovered in 23 episodes (17.2%, 23/134). A total of 143 episodes of IAB, including 17 subsequent episodes, were identified over the study period, representing a cumulative incidence of 4.2 episodes per 1,000 ICU days. Polymicrobial bacteremia was observed in 8 patients (6.3%, 8/126). Primary bacteremia was observed in 41 bacteremic patients (32.5%, 41/126). In 126 patients with IAB, 85 patients developed secondary bacteremia, and the sources of IAB were identified as lungs (61.2%, 52/85), central venous catheter (21.2%, 18/85), urinary tract (16.4%, 14/85) and pleural cavity (1.1%, 1/85). The empiric antimicrobial therapy was appropriate for 69 patients with IAB (54.8%, 69/126).

**Table 1:** Pathogens Associated with the First Episode of IAB in 126 Patients under MV.

Microorganism	Number (%)
Gram-positive microorganisms	
MRSA	16 (11.9)
Enterococcus spp	4 (3.0)
* Streptococcus pneumoniae*	1 (0.7)
Coagulase-negative *staphylococci*	1 (0.7)
Gram-positive bacilli	1 (0.7)
Gram-negative microorganisms	
* Klebsiella pneumoniae*	29 (21.6)
* Pseudomonas aeruginosa*	21 (15.7)
* Elizabethkingia meningoseptica*	18 (13.4)
* Acinetobacter baumannii*	11 (8.2)
* Escherichia coli*	11 (8.2)
* Serratia marcescens*	4 (3.0)
* Burkoderia cepacia*	4 (3.0)
* Pseudomonas putita*	2 (1.5)
* Stenotrophomonas maltophilia*	1 (0.7)
Enterobacter species	3 (2.2)
* Veillonella*	1 (0.7)
* Achromobacter xylosoxidans*	1 (0.7)
* Salmonella enteritis Group D*	1 (0.7)
* Moraxella lacunata*	1 (0.7)
Lactobacillus spp	1 (0.7)
Achromobacter spp	1 (0.7)
GNF-GNB	1 (0.7)
Total	134 (100)

Data are reported as mean ±S.D. or number (%). Eight patients developed polymicrobial bacteremia. GNF-GNB, glucose non-fermenting Gram-negative bacilli; IAB, intensive care unit-acquired bacteremia; MV, mechanical ventilation; MRSA, methicillin-resistant *Staphylococcus aureus*.

### The risk factors for developing IAB

Clinical features of the study patients with and without IAB were presented in **Table 2**. Mechanically ventilated ICU patients who developed IAB were older age (p = 0.038), had higher APACHE II score on ICU admission (p = 0.013), and location prior to ICU admission was mostly nursing homes (p = 0.015) rather than home (p = 0.020), and were significantly associated with the causes of respiratory failure due to chronic obstructive pulmonary disease (COPD) (p = 0.018) and congestive heart failure (CHF) (p<0.001) as compared to those patients without IAB. In the multivariate Cox regression analysis of the potential risk factors for the development of IAB, ventilator support due to COPD (HR 2.057, 95% CI 1.342–3.154; p = 0.001) and CHF (HR 2.028, 95% CI 1.351–3.039; p = 0.001), and patients admitted form nursing home (HR 3.584, 95% CI 1.121–11.494; p = 0.031) remained as the independent risk factors (**Table 3**).

**Table 2:** Clinical Features and Outcomes in the Study Patients with and without IAB.

	All (n = 1453)	IAB (n = 126)	No IAB (n = 1,327)	p
Age* (y)	80 (76–85)	82 (78–85)	80 (75–85)	0.038
Male/female	1104/349	98/28	1006/321	0.664
APACHE II score on ICU admission	18.8±5.4	19.8±5.3	18.7±5.4	0.013
Location prior to ICU admission				
Home	662 (45.6)	42 (33.3)	620 (46.7)	0.020
Ordinary ward	377 (25.9)	39 (31.0)	338 (25.5)	0.202
Intensive care unit	293 (20.2)	25 (19.8)	268 (20.2)	1.000
Local hospital	98 (6.7)	13 (10.3)	85 (6.4)	0.096
Nursing home	12 (0.8)	4 (3.2)	8 (0.6)	0.015
Causes for ventilator support				
Pneumonia	375 (25.8)	30 (23.8)	345 (26.0)	0.670
COPD	283 (19.5)	35 (27.8)	248 (18.7)	0.018
CHF	209 (14.4)	42 (33.3)	167 (12.6)	<0.001
Neurological disease	87 (6.0)	6 (4.8)	81 (6.1)	0.695
Sepsis and septic shock	57 (3.9)	4 (3.2)	53 (4.0)	0.813
Bronchial asthma	29 (2.0)	4 (3.2)	25 (1.9)	0.309
Acute myocardial infarction and CAD	28 (1.9)	0 (0)	28 (2.1)	0.165
Pneumothorax	10 (0.7)	1 (0.8)	9 (0.7)	0.597
ICU stay (d)	23.3±19.8	44.8±26.3	21.3±17.8	<0.001
Total ventilator day (d)	33.3±27.0	55.5±44.8	31.2±25.5	<0.001
Successful weaning	1106 (76.1)	47 (37.3)	1059 (79.8)	<0.001
ICU mortality	230 (15.8)	61 (48.4)	169 (12.7)	<0.001
ICU discharged deposition (n = 1223)				
Ordinary ward	868 (70.9)	48 (73.2)	820 (70.8)	1.000
Home	140 (11.5)	2 (3.6)	138 (11.9)	0.055
Nursing home	83 (6.8)	2 (3.6)	81 (7.0)	0.425
Intensive care unit	32 (2.7)	1 (1.8)	31 (2.7)	1.000
Local hospital	72 (5.9)	7 (10.7)	65 (5.6)	0.141
Respiratory care ward	27 (2.2)	5 (7.1)	22 (1.9)	0.032

Data are reported as mean ±S.D. or number (%). * Values are reported as median (interquartile range). APACHE, Acute Physiology and Chronic Health Evaluation; CAD, coronary artery disease; CHF, congestive heart failure; COPD, chronic obstructive pulmonary disease; IAB, intensive care unit-acquired bacteremia; ICU, intensive care unit.

**Table 3:** Univariate and Multivariate Cox Regression Analysis of Potential Risk Factors for the Development of IAB in the Study Patients (n = 1,453).

	Univariate HR (95% CI)	Multivariate HR (95% CI)
Age	1.000 (0.985–1.016)	-
APACHE II on ICU admission	1.010 (0.977–1.045)	-
Location prior to ICU admission		
Home	0.828 (0.570–1.201)	-
Nursing home	6.802 (2.481–18.518)**	3.584 (1.121–11.494)*
Cause for ventilator support		
COPD	1.557 (1.053–2.304)*	2.057 (1.342–3.154)*
CHF	2.369 (1.631–3.436)**	2.028 (1.351–3.039)*

* p<0.05,

** p<0.001. Multivariate Cox regression performed by the method of forward stepwise technique was applied. APACHE, Acute Physiology and Chronic Health Evaluation; CHF, congestive heart failure; CI, confidence interval; COPD, chronic obstructive pulmonary disease; HR, hazard ratio; IAB, intensive care unit-acquired bacteremia; ICU, intensive care unit.

### IAB and patient outcomes

**Figure 3:**
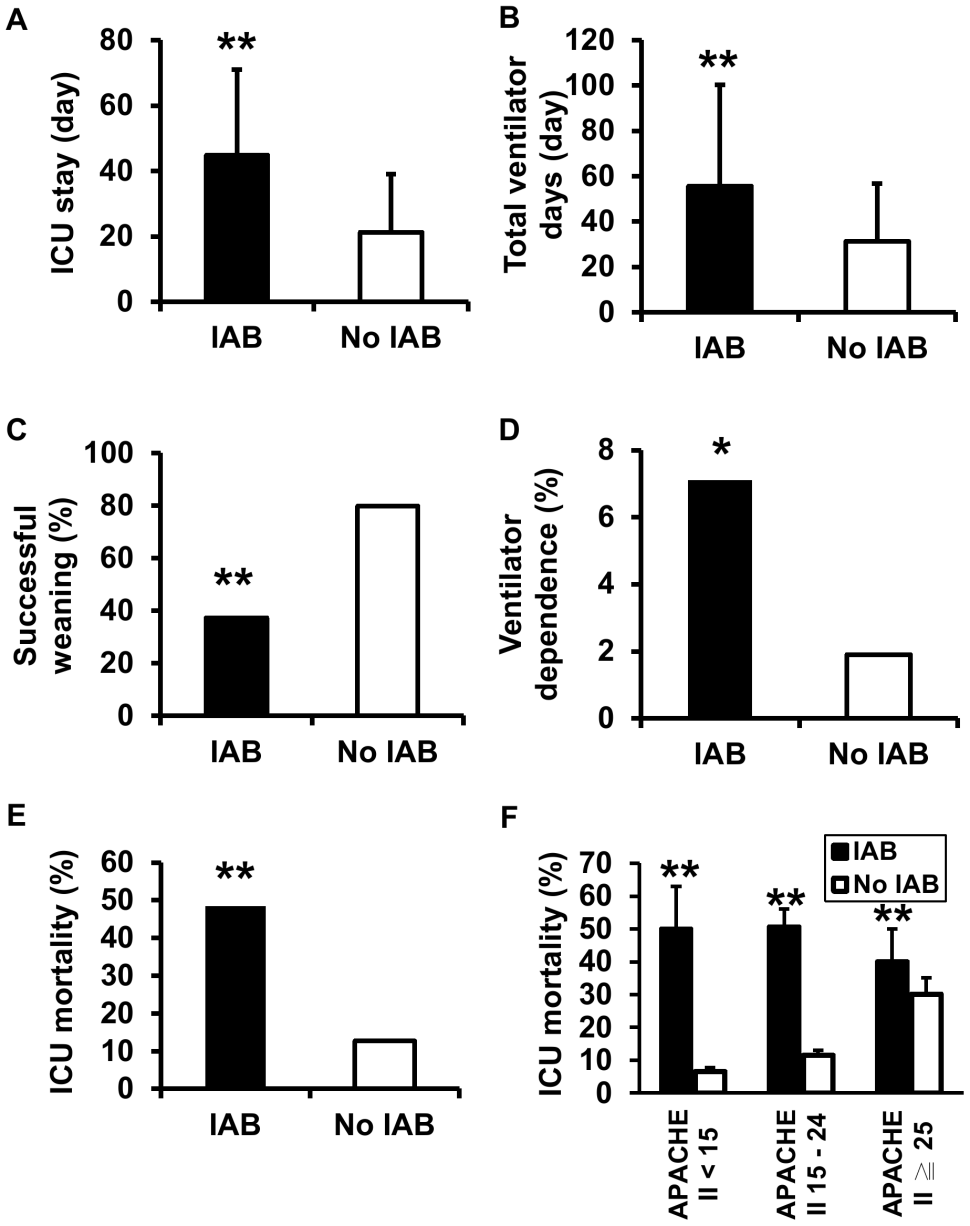
Patient outcomes based on the occurrence of IAB in mechanically ventilated ICU patients. (A–C and E) The numbers of patients in IAB and no IAB groups were n = 126 and n = 1327, respectively. (D) The numbers of patients surviving at ICU discharge in IAB and no IAB groups were n = 65 and n = 1158, respectively. (F) The analysis of ICU mortality was based on APACHE II scores on ICU admission. The numbers of patients in each subgroup were n = 16, n = 299, n = 85, n = 863, n = 25 and n = 165 from left to right, respectively. * denoted p<0.05 and ** denoted p<0.001 for IAB vs. no IAB.

The mechanically ventilated ICU patients who developed IAB were significantly associated with longer length of ICU stay (44.8±26.3 vs. 21.3±17.8 days, p<0.001), prolonged ventilator days (55.5±44.8 vs. 31.2±25.5 days, p<0.001), lower rate of successful weaning (37.3 vs. 79.8%, p<0.001), higher rate of being transferred to respiratory ward due to ventilator dependence (7.1 vs. 1.9%, p = 0.032), and higher ICU mortality rate (48.4 vs. 12.7%, p<0.001) as compared to those patients without IAB (**Table 2** **;** **Figures 3A–E**). The mean values of increase in APACHE II score from ICU admission to IAB development in IAB patients with APACHE II ≧ 25, 15–24 and <15 were 1.5, 6.5 and 10.5, respectively. Moreover, IAB significantly contributed to the higher rate of ICU mortality across a wide range of APACHE II score (**Figure 3F**). The results of multivariate Cox regression analysis showed that APACHE II on ICU admission and IAB were the independent risk factors for ICU mortality (HR 1.056, 95% CI 1.028–1.084; p<0.001 and HR 1.510, 95% CI 1.054–1.123; p = 0.010, respectively) (**Table 4**).

**Table 4:** Univariate and Multivariate Cox Regression Analysis of Potential Risk Factors for ICU Mortality in the Study Patients (n = 1,453).

	Univariate HR (95% CI)	Multivariate HR (95% CI)
Patient admitted from ordinary ward	0.818(0.620–1.080)	-
Age	1.002 (0.999–1.014)	-
APACHE II on ICU admission	1.056 (1.029–1.084)**	1.056 (1.028–1.084)**
IAB	1.490 (1.101–2.020)**	1.510 (1.054–1.123)*

*p<0.05,

**p<0.001. Multivariate Cox regression performed by the method of forward stepwise technique was applied. APACHE, Acute Physiology and Chronic Health Evaluation; CI, confidence interval; HR, hazard ratio; IAB, intensive care unit-acquired bacteremia; ICU, intensive care unit.

### Baseline characteristics of the IAB patients based on specific bacterial species

The baseline characteristics of IAB based on the most common pathogens are shown in **Table 5**. IAB due to *Klebsiella pneumoniae* and *Pseudomonas aeruginosa* mostly originated from the lungs (p = 0.031 and p<0.001 respectively). IAB due to *Escherichia coli* frequently originated from the urinary tract (p = 0.020) and methicillin-resistant *Staphylococcus aureus* (MRSA) was significantly related to the infection source of CVC (p = 0.002). IAB due to *Elizabethkingia meningoseptica* was mostly classified as primary bacteremia (p = 0.002). The patients with IAB due to *P. aeruginosa* were more likely polymicrobial (p = 0.019) and were significantly associated with prior antibiotic use (p = 0.008) as compared to patients with IAB due to non-*P. aeruginosa*. Patients with IAB due to *E. meningoseptica* and MRSA significantly showed multiple sets of positive blood culture compared to those with IAB due to non-*E. meningoseptica* and non-MRSA (p = 0.001 and p = 0.048 respectively). IAB due to *E. coli* significantly occurred earlier than IAB due to non-*E. coli* (p = 0.012).

**Table 5:** Baseline Characteristics of Study Patients with IAB Based on Bacterial Species.

	*K. pneumonia* (n = 29)	*P. aeruginosa* (n = 21)	*E. meningoseptica* (n = 18)	*A. baumannii* (n = 11)	*E. coli* (n = 11)	MRSA (n = 16)	P
Age* (y)	81 (76–86)	82 (78–89)	82 (78–88)	81 (66–86)	82 (75–90)	82 (79–84)	0.821
APACHE II score on ICU admission	19.5±5.2	19.0±4.6	17.6±5.3	21.8±6.0	17.6±3.3	20.9±4.8	0.181
Comorbidity							
Active malignancy	4 (13.8)	2 (9.5)	2 (11.1)	1 (9.1)	1 (9.1)	1 (6.3)	0.589
COPD	14 (48.3)	13 (61.9)	11 (61.1)	3 (27.3)	4 (36.4)	6 (37.5)	0.020
CHF	8 (27.6)	7 (33.3)	8 (44.4)	6 (54.5)	2 (18.2)	7 (43.8)	0.549
Diabetes mellitus	9 (31.0)	6 (28.6)	6 (33.3)	5 (45.5)	3 (27.3)	5 (31.3)	0.637
Primary bacteremia	2 (6.9) a	0 (0) b	12 (66.7) c	4 (36.4)	6 (54.5)	4 (25.0)	0.032
Sources of pathogens							
Lungs	17 (58.6)^a^	17(81.0) b	5 (27.8)	6 (54.5)	1 (9.1)^d^	4 (25.0)	0.000
CVC	6 (20.7)	0 (0) c	1 (5.6)	0 (0)	0 (0)	7 (43.8) e	0.109
Urinary tract	4 (13.8)	3(14.3)	0 (0)	1 (9.1)	4 (36.4) d	0 (0)	0.572
Empyema	0 (0)	1 (4.8)	0 (0)	0 (0)	0 (0)	1 (6.3)	1.000
Multiple sets of positive hemoculture	3 (10.3)	3 (14.3)	10 (55.6) c	2 (18.2)	2 (18.2)	7 (43.8) e	0.036
Polymicrobial pathogens	5 (17.2)	5 (23.8) b	2 (11.1)	1 (9.1)	1 (9.1)	1 (6.3)	0.168
Prior antibiotic use	11 (37.9)	15 (71.4) b	5 (27.8)	5 (45.5)	4 (36.4)	7 (43.8)	0.037
Duration of ICU admission until IAB (d)	22.0±15.8	20.1±16.0	21.3±16.8	14.8±10.4	10.2±6.5 d	17.0±14.9	0.187

Data are reported as mean ±S.D. or number (%). *Values were reported as median (interquartile range). Nonparametric Chi-Square test and Kruskal-Wallis test were used for the six most common pathogens of IAB to compare categorical and continuous variables, respectively, and p values are depicted in the Table. The Chi-Square test/Fisher exact test and Mann-Whitney U test were used for categorical and continuous variables, respectively, to identify the significant difference between patients harboring the individual pathogen versus patients not harboring this pathogen. APACHE, Acute Physiology and Chronic Health Evaluation; CHF, congestive heart failure; COPD, chronic obstructive pulmonary disease; CVC, central venous catheter; IAB, ICU-acquired bacteremia; ICU, intensive care unit; MRSA, methicillin-resistant *Staphylococcus aureus*.

a p<0.05, IAB due to *K. pneumoniae* vs. non-*K. pneumoniae*;

b p<0.05, IAB due to *P. aeruginosa* vs. non-*P. aeruginosa*;

c p<0.05, IAB due to *E. meningoseptica* vs. non-*E. meningoseptica*;

d p<0.05, IAB due to *E. coli* vs. non-*E. coli*;

e p<0.05, IAB due to MRSA vs. non-MRSA.

### Clinical characteristics at the onset of IAB based on specific bacterial species

Clinical characteristics at the onset of IAB and patient outcomes in IAB patients based on the six common pathogens are shown in **Table 6**. The patients with IAB due to *P. aeruginosa* had higher serum levels of blood urea nitrogen (BUN) (114.3±63.7 vs. 94.8±50.9 mg/dl, p = 0.030) and greater numbers of organ dysfunction (1.6±1.4 vs. 0.9±1.1, p = 0.040) as compared to those patients with IAB due to non-*P. aeruginosa*. IAB due to *E. meningoseptica* was significantly associated with a lower rate of effective empiric antibiotic (16.7 vs. 61.1%, p = 0.001), and the increased time delay until appropriate antibiotics (77.5±47.8 vs. 33.8±37.8 hours, p<0.001). IAB due to *E. meningoseptica* was significantly associated with lower serum levels of BUN (60.9±25.6 vs. 92.8±56.5 mg/dl, p = 0.024), lower rate of shock (11.1 vs. 39.3%, p = 0.030) and less need for acute hemodialysis (0 vs. 19.4%, p = 0.041) as compared to IAB due to non-*E. meningoseptica*. IAB due to *Acinetobacter baumanii* seemed to have better outcomes, including greater 28-day ventilator-free days (6.7±8.3 vs. 3.6±7.2 days, p = 0.048) and lower rate of ICU mortality (9.1 vs. 51.7%, p = 0.017) as compared to IAB due to non-*A. baumanii*.

**Table 6:** Clinical Features at the Onset of IAB in Study Patients with IAB Based on Bacterial Species.

	*K. pneumonia* (n = 29)	*P. aeruginosa* (n = 21)	*E. meningoseptica* (n = 18)	*A. baumannii* (n = 11)	*E. coli* (n = 11)	MRSA (n = 16)	P
APACHE II	24.4±6.7	27.7±6.9	22.8±5.8	26.7±5.6	22.4±7.2	26.3±8.6	0.235
Oxygen ratio (mmHg)	244.2±163.7	220.6±135.1	275.9±182.5	245.4±116.5	208.5±101.3	243.8±134.8	0.922
BUN (mg/dl)	78.0±52.8	114.3±63.7 a	60.9±25.6 b	105.0±72.7	87.4±62.5	89.3±54.3	0.095
Creatinine (mg/dl)	2.1±1.5	2.4±1.5	1.6±1.0	2.5±1.5	2.2±2.0	2.6±1.4	0.466
Effective empiric antibiotic	15 (51.7)	14 (66.7)	3 (16.7)^b^	7 (63.6)	6 (54.5)	8 (50.0)	0.028
Time delay until appropriate antibiotic used (h)	37.8±42.4	24.0±30.9	77.5±47^b^	46.2±49.4	34.4±32.8	38.1±36.9	0.024
Shock	10 (34.5)	9 (42.9)	2 (11.1)^b^	5 (45.5)	2 (18.2)	4 (25.0)	0.049
Need for acute hemodialysis	5 (17.2)	5 (23.8)	0 (0)^b^	1 (9.1)	0 (0)	4 (25.0)	0.413
Organ dysfunction (No.)	0.8±1.0	1.6±1.4 a	0.5±0.5	0.9±1.1	0.9±0.9	1.2±1.2	0.152
ICU stay (d)	54.6±37.0	38.4±24.0	49.4±25.9	41.9±15.7	41.7±26.3	34.1±19.6	0.253
Successful weaning	10 (34.5)	6 (28.6)	9 (50.0)	6 (54.5)	5 (45.5)	9 (56.3)	0.721
28-day ventilator-free days (d)	3.0±7.1	3.1±7.2	2.8±6.7	6.7±8.3 c	6.8±9.6	6.0±8.0	0.102
ICU mortality	15 (51.7)	12 (57.1)	10 (55.6)	1 (9.1)^c^	3 (27.3)	7 (43.8)	0.003

Data are reported as mean ±S.D. or number (%). Nonparametric Chi-Square test and Kruskal-Wallis test were used for the six most common pathogens of IAB to compare categorical and continuous variables, respectively, and p values are depicted in the table. The Chi-Square test/Fisher exact test and Mann-Whitney U test were used for categorical and continuous variables, respectively, to identify the significant difference between patients harboring the individual pathogen versus patients not harboring this pathogen. APACHE, Acute Physiology and Chronic Health Evaluation; BUN, blood urea nitrogen; ICU, intensive care unit; MRSA, methicillin-resistant *Staphylococcus aureus*.

a p<0.05, IAB due to *P. aeruginosa* vs. non-*P. aeruginosa*;

b p<0.05, IAB due to *E. meningoseptica* vs. non-*E. meningoseptica*;

c p<0.05, IAB due to *A. baumannii* vs. non-*A. baumannii*.

## Discussion

Bacteremia is one of the most common nosocomial infections and is harmful to critically ill patients [6], [19]–[21]. In the current study, we found that mechanically ventilated ICU patients with IAB compared to those without IAB had longer length of ICU stay, prolonged ventilator use, lower rate of successful weaning, and higher rate of ventilator dependence. Importantly, IAB was considered as the independent risk factor for ICU mortality and was associated with four-fold ICU mortality risk in comparison to those patients without IAB. We further identified the independent risk factors for developing IAB in mechanically ventilated ICU patients as being on ventilator support due to COPD and CHF, and patients admitted from nursing home. We also described the clinical characteristics related to six specific bacterial species. To our knowledge, our study is the first to describe the clinical features and outcomes of IAB in mechanically ventilated ICU patients, and to report the clinical characteristics of IAB related to specific bacterial species. The results of our study can be utilized by intensivists to refine the management of IAB and may improve the quality of care in mechanically ventilated patients in the ICU setting.

The incidence of IAB in mechanically ventilated patients has not yet been determined. Here, we report that the cumulative incidence of IAB in ICU patients with MV was 4.2 episodes per 1,000 ICU days; that is lower than in previous studies showing a rate of 5.2 to 18.9 episodes per 1,000 ICU days in critically ill patients with and without MV [10], [20], [21]. The discrepancies may be due to the difference in the causative pathogens acquired in the ICUs between mechanically ventilated and non-mechanically ventilated critically ill patients. For example, infection by gram-negative microorganisms (83%) was dominant in our study, but the majority of IAB cases were attributed to gram-positive pathogens (73%) in the Laupland study [10]. Moreover, the median length of ICU stay in our study enrolling mechanically ventilated patients was also longer than that in a previous study (17.5 vs. 4.9 days) [10]. Older patient age in our study compared with that in other studies was also observed [10], [20], [21]. In our study, approximately one half of enrolled patients had a delay in recovering from medical critical conditions and were referred from other ICUs and ordinary wards. The patient sources in this study were different from those in other studies conducted in mixed ICUs with medical and surgical patients enrolled. The difference of patient sources may be associated with the discrepancy of age and IAB-related characteristics. These factors might be the reason for the low incidence of IAB in ICU patients under MV.

Nosocomial infections in ICU are associated with high mortality and medical expenditure [1]–[5]. In critically ill patients developing IAB, Laupland et al. reported a 2-fold increase in ICU mortality [10]. In our study, we demonstrated a 4-fold increase in ICU mortality in ICU patients under MV who developed IAB as compared to those who did not. We further analyzed the impact of IAB on the ICU mortality in the subgroup of patients with APACHE II score <15, 15–24 and ≧ 25. The results showed that IAB led to a greater increase in APACHE II score in IAB patients with admitted APACHE II <15 compared to other subgroups and was significantly associated with the increase of ICU mortality across the wide range of disease severities. Importantly, in the subgroups with less serious severity of disease (APACHE II <15 and 15–24), excess ICU mortality of approximately 40% was observed in patients with IAB as compared to those without IAB. Our study showed that IAB not only had an impact on patient survival but also hampered the discontinuation of ventilator in mechanically ventilated patients in the ICU setting.

It is important to identify risk factors for intensivists to manage nosocomial infections early in the ICU setting. Several risk factors for the development of IAB, including younger age, immune-compromised status, diabetes mellitus, admission to ICU, higher therapeutic intervention scoring system scores, higher admitting APACHE II, and increased length of ICU stay, have been mentioned in critically ill patients [10], [20], [22]. In ICU patients with MV, we identified older age as the risk factor for IAB that was different from the population-based assessment of IAB from Laupland's study [10]. Severe disease measured by the admitting APACHE II score was considered as a risk factor for IAB in our study and others [10], [20]. We also reported that in ICU patients under MV, patient admitted from nursing homes, not home, was associated with a higher risk of developing IAB. Notably, in the multivariate Cox regression analysis, we identified ventilator support for COPD and CHF, and patients admitted from nursing home as the independent risk factors for developing IAB. Although patients with respiratory failure due to pneumonia and sepsis were potentially associated with developing bacteremia simultaneously, both of those acute illnesses seemingly had less influence on the bacteremia development after ICU admission. The above-mentioned information enables intensivists to become more aware of the mechanically ventilated patients who are at the risk of developing IAB and initiate the treatment of IAB early in the ICU setting.

One of the important findings in our study was the clinical characteristics related to specific bacterial species. We identified that IAB due to *P. aeruginosa* was significantly associated with prior use of antibiotics, and the lung was the most likely entry site with polymicrobial infection. These patients were frequently implicated with distal organ dysfunction, and in particular, renal failure. Other investigators also report that IAB due to *P. aeruginosa* is more likely to be nonrespondent on the basis of continued need for vasopressor support [23], [24]. It is noted that *E. meningoseptica* was one of the most commonly identified pathogens in our study. We observed that patients with IAB due to *E. meningoseptica* presented with milder illness in general but longer duration of delayed appropriate antibiotic treatment as compared to those with IAB due to *P. aeurginosa*. The inadequate and/or delayed appropriate antibiotic treatment may potentially have increased the risk of death in patients with *E. meningoseptica* bacteremia and led to a similar rate of ICU mortality between IAB due to *E. meningoseptica* and *P. aeurginosa*. [25]–[27]. Another interesting finding of our study is that patients with IAB due to *A. baumannii* had better outcomes, with a lower ICU mortality rate and shorter ventilator days. This observation may be explained by the fact that most *A. baumannii* causing IAB (72.7%) were not multiple-drug resistant [28]; therefore, these patients generally responded well to antibiotic therapy. Urinary tract infection is one of the important nosocomial infections, and *E. coli* is reported to be the most common pathogen that potentially causes the development of IAB in the ICU [29]. In our study, IAB due to *E. coli* mostly originated from the urinary tract and significantly developed earlier (mean time of 10 days after ICU admission). In our study, MRSA was the most common gram-positive bacteria for IAB in mechanically ventilated ICU patients and was mostly related to central catheter infection. Kullar et al. also report that catheter-related infections and endocarditis are the most common sites of MRSA bacteremia for older (65 years and older) and younger (less than 65 years) adults, respectively [30]. We also identified that IAB due to MRSA was associated with multiple sets of positive hemoculture and made necessary immediate removal of the CVC.

There are several limitations in our study. The study was conducted in a respiratory ICU, where gram-negative microorganisms were the main pathogens of IAB. The patients enrolled were elderly and ventilated for a long period. This overall picture of IAB may be different in a general multidisciplinary ICU containing a mix of medical and surgical patients. Second, the patients were followed until ICU discharge; thus, the impact of IAB on long-term outcomes was not evaluated. Third, the study was retrospective and observational, and we did not prospectively evaluate, assess and examine the prevalence of bacteremia under a standardized protocol for all critically ill patients, independent of the underlying diagnoses and the application of mechanical ventilation.

## Conclusions

We reported that IAB was the independent risk factor for ICU mortality in mechanically ventilated ICU patients and was significantly associated with longer length of ICU stay, prolonged ventilator days, lower rate of successful ventilator weaning, and higher rate of ventilator dependency. The risk factors for developing IAB and the clinical characteristics related to specific bacterial species identified in our study may help intensivists develop the appropriate strategies to refine the management of IAB in mechanically ventilated patients in the ICU setting.
